# Late Cardiac Toxic Effects Associated With Treatment Protocols for Hodgkin Lymphoma in Children

**DOI:** 10.1001/jamanetworkopen.2023.51062

**Published:** 2024-01-19

**Authors:** Andrea C. Lo, Amy Liu, Qi Liu, Yutaka Yasui, Sharon M. Castellino, Kara M. Kelly, Alex F. Hererra, Jonathan W. Friedberg, Debra L. Friedman, Cindy L. Schwartz, Qinglin Pei, Sandy Kessel, Samuel Bergeron-Gravel, Hitesh Dama, Kenneth Roberts, Louis S. Constine, David C. Hodgson

**Affiliations:** 1 Department of Radiation Oncology, BC Cancer, University of British Columbia, Vancouver, British Columbia, Canada; 2 Dalla Lana School of Public Health, University of Toronto, Toronto, Ontario, Canada; 3 Department of Public Health Sciences, University of Alberta, Edmonton, Alberta, Canada; 4 Epidemiology and Cancer Control Department, St Jude Children’s Research Hospital, Memphis, Tennessee; 5 Aflac Cancer and Blood Disorders Center, Children’s Healthcare of Atlanta, Atlanta, Georgia; 6 Department of Pediatric Oncology, Roswell Park Cancer Institute and Oishei Children’s Hospital, University at Buffalo School of Medicine and Biomedical Sciences, Buffalo, New York; 7 Department of Hematology, City of Hope, Duarte, California; 8 Department of Medical Oncology, University of Rochester, Rochester, New York; 9 Division of Pediatric Hematology/Oncology, Vanderbilt-Ingram Cancer Center, Vanderbilt University Medical Center, Nashville, Tennessee; 10 Department of Pediatrics, Children’s Hospital of Wisconsin, Medical College of Wisconsin, Milwaukee; 11 Children’s Oncology Group, Statistics and Data Center, Department of Biostatistics, University of Florida, Gainesville; 12 Imaging and Radiation Oncology Core, Lincoln, Rhode Island; 13 Centre Hospitalier Universitaire de Québec–Université Laval, Québec, Quebec, Canada; 14 Princess Margaret Cancer Centre, Toronto, Ontario, Canada; 15 Therapeutic Radiology, Yale University School of Medicine, New Haven, Connecticut; 16 Department of Radiation Oncology, Wilmot Cancer Institute, University of Rochester, Rochester, New York; 17 Department of Radiation Oncology, Princess Margaret Cancer Centre–University Health Network, Toronto, Ontario, Canada

## Abstract

**Importance:**

Contemporary North American trials for children with Hodgkin lymphoma (HL) have decreased radiation therapy (RT) use and increased pharmacologic cardioprotection but also increased the cumulative doxorubicin dose, making overall treatment consequences for late cardiac toxic effects uncertain.

**Objective:**

To estimate the risk of cardiac toxic effects associated with treatments used in modern pediatric HL clinical trials.

**Design, Setting, and Participants:**

For this cohort study, Fine and Gray models were fitted using survivors in the Childhood Cancer Survivor Study who were diagnosed with HL between January 1, 1970, and December 31, 1999, and were followed for a median of 23.5 (range, 5.0-46.3) years. These models were applied to the exposures in the study population to estimate the 30-year cumulative incidence of cardiac disease. The study population comprised patients with intermediate-risk or high-risk HL treated in 4 consecutive Children’s Oncology Group clinical trials from September 2002 to October 2022: AHOD0031, AHOD0831, AHOD1331, and S1826. Data analysis was performed from April 2020 to February 2023.

**Exposures:**

All patients received chemotherapy including doxorubicin, and some patients received mediastinal RT, dexrazoxane, or mediastinal RT and dexrazoxane.

**Main Outcomes and Measures:**

Estimated 30-year cumulative incidence of grade 3 to 5 cardiac disease.

**Results:**

The study cohort comprised 2563 patients, with a median age at diagnosis of 15 (range, 1-22) years. More than half of the patients were male (1357 [52.9%]). All 2563 patients received doxorubicin, 1362 patients (53.1%) received mediastinal RT, and 307 patients (12.0%) received dexrazoxane. Radiation therapy use and the median mean heart dose among patients receiving RT decreased, whereas the planned cumulative dose of doxorubicin and use of dexrazoxane cardioprotection increased. For patients treated at age 15 years, the estimated 30-year cumulative incidence of severe or fatal cardiac disease was 9.6% (95% CI, 4.2%-16.4%) in the AHOD0031 standard treatment group (enrolled 2002-2009), 8.6% (95% CI, 3.8%-14.9%) in the AHOD0831 trial (enrolled 2009-2012), 8.2% (95% CI, 3.6%-14.3%) in the AHOD1331 trial (enrolled 2015-2019), and 6.2% (95% CI, 2.7%-10.9%) in the S1826 trial (enrolled 2019-2022), whereas the expected rate in an untreated population was 5.0% (95% CI, 2.1%-9.3%). Despite the estimated reduction in late cardiac morbidity, the frequency of recommended echocardiographic screening among survivors will increase based on current guidelines.

**Conclusions and Relevance:**

In this cohort study of sequential HL trials, reductions in the proportion of children receiving mediastinal RT and increases in dexrazoxane use were estimated to offset the increased doxorubicin dose and produce a net reduction in late cardiac disease. Further studies on dexrazoxane are warranted to confirm whether its role in reducing cardiac toxic effects is maintained long term. These findings suggest that survivorship follow-up guidelines should be refined to align with the risks associated with treatment.

## Key Points

**Question:**

What is the risk of cardiac toxic effects associated with therapies used in pediatric Hodgkin lymphoma (HL) trials?

**Findings:**

In this cohort study of 2563 patients with HL treated in 4 consecutive Children’s Oncology Group clinical trials between 2002 and 2022, the 30-year cumulative incidence of grade 3 to 5 cardiac disease was estimated to decrease from 10% in the first trial to 6% in the last trial.

**Meaning:**

These findings suggest that evolutions in HL treatment are associated with a net reduction in late cardiac disease.

## Teaser

This cohort study estimates the risk of cardiac toxic effects associated with treatments used in 4 consecutive pediatric Hodgkin lymphoma clinical trials.

## Introduction

The elevated risk of delayed morbidity caused by Hodgkin lymphoma (HL) treatment is well documented and has motivated efforts in HL clinical trials to reduce exposure to the responsible components of treatment, particularly radiation therapy (RT).^1,2,3^ Cardiac morbidity among survivors of childhood HL is one of the more serious adverse outcomes of treatment; however, it is uncertain to what extent changes in HL protocols are likely to reduce the risk of late cardiac morbidity or simplify the follow-up of long-term survivors. The magnitude of potential benefit to further modifications of these protocols also is unknown.^3,4^

A quantitative understanding of the extent to which modifying HL treatment reduces the risk of late toxic effects is important for several reasons. First, because a reduction of treatment intensity comes as a trade-off against a potential increase in relapse risk, making rational decisions about this trade-off requires an understanding of the size of the expected benefit. Second, quantitative estimates of the contribution of different components of treatment to late toxicity risk can facilitate better decisions about which future treatment modifications will have the greatest role in further reducing late morbidity. Finally, recommendations for survivorship care are based primarily on treatment exposures, and the lifelong experience of survivors of childhood cancer requires consideration of the burden of follow-up care.

Relying exclusively on observational studies to guide rational treatment reduction strategies is problematic because direct observation of cardiac late effects from modern HL therapy will not be possible for several decades. However, there is sufficient long-term experience with the 2 most cardiotoxic agents: RT and doxorubicin. Robust models of late cardiac risk are available that describe the dose-risk association of these agents among childhood cancer survivors.^5,6^ We tailored these models to apply to patients with HL treated in recent Children’s Oncology Group (COG) trials, with the aims (1) to determine whether late cardiac risk can be expected to decrease as trials have evolved and (2) to identify further interventions that are likely to produce the most benefit. We hypothesized that the late cardiac risk associated with HL therapy will decrease over time, with dexrazoxane being an important contribution to this risk reduction.

## Methods

The study cohort comprised participants in 4 COG trials enrolling patients with intermediate-risk and high-risk HL: AHOD0031, AHOD0831, AHOD1331, and S1826. Local institutional review boards approved the trials and informed consent was obtained as described later in this section. The trials were conducted in accordance with the Declaration of Helsinki.^7^ This study followed the Strengthening the Reporting of Observational Studies in Epidemiology (STROBE) reporting guideline.

Trial AHOD0031 (accrual 2002-2009) randomized patients diagnosed with classical HL (at age <22 years) with stage IB, IAE, IIB, IIAE, IIIA, IVA with or without bulk disease, or IA or IIA with bulk disease.^8^ All patients received 4 cycles of doxorubicin, bleomycin, vincristine, etoposide, prednisone, and cyclophosphamide (ABVE-PC) with a cumulative doxorubicin dose of 200 mg/m^2^. Those who had a slow early response to the first 2 cycles of ABVE-PC were randomized to receive vs not receive 2 cycles of dexamethasone, etoposide, cytarabine, and cisplatin. With regard to RT, patients were randomized to response-adapted (experimental) vs risk-adapted (standard) therapy. In the experimental treatment group, patients with a slow early response received involved-field radiation therapy (IFRT) to 21 Gy, whereas those with both a rapid early response and complete response did not receive IFRT. In the standard treatment group, all patients received 21 Gy of IFRT regardless of early response.

Trial AHOD0831 (accrual 2009-2012) included patients diagnosed (at age <22 years) with stage IIIB or stage IVB classical HL^9^ and treated with ABVE-PC to a cumulative doxorubicin dose of 200 mg/m^2^. Patients with a slow early response received an additional 2 cycles of ifosfamide-vinorelbine and IFRT to 21 Gy to sites of slow early response, bulk at diagnosis, or both.

Trial AHOD1331 (accrual 2015-2019) randomized patients diagnosed (at age <22 years) with stage IIB with bulk, IIIB, IVA, or IVB classical HL to brentuximab vedotin-AVE-PC vs ABVE-PC.^10^ The cumulative doxorubicin dose was 250 mg/m^2^, and 21 to 30 Gy of involved-site RT was prescribed to sites of slow early response, mediastinal bulk at diagnosis, or both.

Trial S1826 (accrual 2019-2022) randomized patients diagnosed (at age ≥12 years) with stage III and IV classical HL to doxorubicin, vinblastine, and dacarbazine (AVD) plus nivolumab vs AVD plus brentuximab vedotin, with a cumulative doxorubicin dose of 300 mg/m^2^. Patients received residual disease RT to 30 to 36 Gy if they met all of the following criteria: (1) a Deauville score of 4 or 5, (2) a residual nodal mass of 2.5 cm or greater in axial diameter, and (3) a 30% or greater reduction in maximum transverse diameter compared with pretreatment imaging.

Data collection for the COG trials occurred at each participating institution after approval by local institutional review boards in accordance with institutional policies. Written informed consent was obtained from all patients aged 18 years or older; for all patients aged younger than 18 years, written informed consent was obtained from a parent or guardian, with assent obtained from the child or adolescent. The Childhood Cancer Survivor Study (CCSS) methods and study design have previously been described in detail.^11^ Data on race and ethnicity are included in CCSS studies to assess health disparities and inequities in participation and outcomes and they are obtained from self-report questionnaires; however, complete race and ethnicity data were not available for all 4 COG trials in this analysis.

For children enrolled at COG sites who received RT, the RT plans were submitted to the Imaging and Radiation Oncology Core in Lincoln, Rhode Island, in electronic Digital Imaging and Communications in Medicine (DICOM) format starting in 2006. Normal organs at risk, including the heart, were contoured by the treating radiation oncologist. For the current study, DICOM-RT files for patients receiving thoracic RT were retrieved from the Imaging and Radiation Oncology Core and cardiac contours were reviewed by 1 of 3 radiation oncologists (A.L., S.B.-G., or D.C.H.) to ensure consistency with cardiac contouring guidelines of the Radiation Therapy Oncology Group.^12^ For each patient receiving mediastinal RT who had DICOM-RT files available, the mean heart dose, which is the average dose received by the whole heart volume, was acquired. For each protocol, the mean value of these individual-level mean heart doses was used in the estimates of cardiac risk.

### Statistical Analysis

Descriptive statistics were used to summarize patient characteristics. Comparisons of the proportion of patients receiving mediastinal RT and the mean heart dose among trials were performed using χ^2^ and Kruskal-Wallis tests, respectively.

Creation of the models that describe the association among radiation dose, doxorubicin dose, and cardiac risk in the CCSS cohort has been detailed previously.^5^ For this study, Fine and Gray models quantifying the association among age, sex, doxorubicin dose, alkylating agent exposure, and cardiac radiation dose and late cardiac risk were fitted specifically to the HL survivors in the CCSS cohort. As defined by the CCSS, cardiac disease was restricted to grade 3 to 5 cardiac conditions, including coronary artery disease (CAD), heart failure (HF), valvular disease, pericardial disease, and arrhythmias.^8^ Noncardiac death was considered as a competing risk event for the 3 outcomes (any cardiac disease, CAD, and HF). Death from cardiac disease was coded as an event. A 5-year survival of 97.1% for HL survivors diagnosed at age 0 to 19 years from 2002 to 2016 was applied.^13^ Model estimates were used to project cumulative incidences of each outcome for COG trial patients, accounting for statistically significant treatment and demographic characteristics. Model inputs related to treatment exposures (ie, cumulative doxorubicin dose, mediastinal RT use, mean heart dose, and dexrazoxane use) were determined from COG trial databases for the AHOD0031, AHOD0831, AHOD1331, and S1826 completed trials. Based on trial results, we modeled the AHOD0031 standard treatment group with 85% mediastinal RT use and the AHOD0031 experimental treatment group with 40% mediastinal RT use. To obtain 95% CIs, 1000 bootstrapped model estimates and cumulative incidence curves were generated to calculate the 2.5th and 97.5th percentiles. To reflect outcomes of dexrazoxane treatment, a 70% reduction was applied in the projected cumulative incidences for patients who received both doxorubicin and dexrazoxane, compared with those who did not, based on prior results among childhood cancer survivors.^14^

To calculate the cumulative incidence for any cardiac disease, CAD, and HF for the average 15-year-old patient in each trial, weighting of the cumulative incidence function was performed according to the average mean heart dose and cumulative doxorubicin dose and incorporated the likelihood that the patient received mediastinal RT and dexrazoxane in the respective trial. To account for variation in the age distribution of enrolled patients in different trials, an age at treatment of 15 years was used for the base models, as this was the median age in the AHOD0031, AHOD0831, and AHOD1331 trials. Results for the average 15-year-old patient can be considered equivalent to a cohort of 15-year-old patients with an equal number of males and females enrolled. Sex- and race and ethnicity–specific outcomes were also estimated.

We based the proportion of HL survivors who will be recommended to have echocardiographic screening on version 5 (2018) of the COG long-term follow-up guidelines for survivors of childhood, adolescent, and young adult cancers.^15^ SAS, version 9.4 (SAS Institute), and R, version 4.3.0 (R Project for Statistical Computing), were used for statistical analyses. All tests were 2 sided, and *P* < .05 indicated statistical significance. Data analysis was performed from April 2020 to February 2023.

## Results

The study cohort included 3320 eligible patients enrolled in the 4 trials, with a median age at diagnosis of 15 (range, 1-22) years. Of the 2563 patients included in the analysis, 1357 (52.9%) were male and 1206 were female (47.1%). Table 1 displays the demographic characteristics and treatment exposures of the study cohort. The number of patients receiving mediastinal RT decreased significantly over the course of the 4 trials, from 972 (61.8%) to 1 (0.4%; *P* < .001), as did the mean (SD) mean heart dose among those receiving mediastinal RT (13.2 [5.7] to 3.8 [not applicable] Gy; *P* < .001). The cumulative dose of doxorubicin increased from 200 mg/m^2^ in the AHOD0031 trial to 300 mg/m^2^ in the more recent S1826 trial.

**Table 1.** Demographic Characteristics and Treatment Exposures of the Study Cohort^a^

Variable	Children’s Oncology Group trial (N = 2563)				
	AHOD0031 (n = 1574)	AHOD0831 (n = 165)	AHOD1331 (n = 587)	S1826 pediatric cohort (n = 237)
Age at treatment, median (range), y	15 (1-21)	15 (1-21)	15 (3-21)	16 (12-18)
Sex				
Male	826 (52.5)	101 (61.2)	311 (53.0)	119 (50.2)
Female	748 (47.5)	64 (38.8)	276 (47.0)	118 (49.8)
Treatment received in addition to doxorubicin				
Mediastinal RT	972 (61.8)	95 (57.6)	294 (50.1)	1 (0.4)
Dexrazoxane	0	8 (4.8)	111 (18.9)	188 (79.3)
Exposure analysis of the mediastinal RT group (n = 459)				
No. of patients receiving mediastinal RT	72	88	298	1
Year of RT	2002-2009	2009-2012	2015-2019	2019-2022
Mean heart dose, Gy				
Mean (SD)	13.2 (5.7)	13.6 (4.7)	10.0 (4.0)	3.8 (NA)
Median (range)	15.0 (0.4-22.0)	14.2 (0.7-22.0)	10.0 (0.1-20.5)	3.8 (NA)
Heart dose group, Gy				
<5	7 (9.7)	4 (4.5)	39 (13.1)	1 (100)
5 to <10	15 (20.8)	17 (19.3)	106 (35.6)	0
10 to <15	14 (19.4)	28 (31.8)	125 (41.9)	0
15 to <23	36 (50.0)	38 (43.2)	24 (8.1)	0
Cumulative anthracycline dose, mg/m^2^	200	200	250	300

Abbreviations: NA, not applicable; RT, radiation therapy.

^a^ Values are presented as No. (%) of patients unless indicated otherwise.

Table 2 shows the subdistribution hazard ratios (HRs) for grade 3 to 5 cardiac disease from Fine and Gray models fitted to the HL survivors in the CCSS cohort. A greater mean cardiac RT dose was associated with an increased risk of any cardiac disease (HR, 1.40 per 10 Gy [95% CI, 1.26-1.55]; *P* < .001), CAD (HR, 1.43 per 10 Gy [95% CI, 1.26-1.62]; *P* < .001), and HF (HR, 1.59 per 10 Gy [95% CI, 1.36-1.86]; *P* < .001), whereas a greater cumulative doxorubicin dose only increased the risk of HF (HR, 1.49 per 100 mg/m^2^ [95% CI, 1.31-1.69]; *P* < .001). Male patients had a higher risk of CAD and HF than female patients (HR, 1.72 [95% CI, 1.32-2.26]; *P* < .001), and the risk of any cardiac disease and CAD increased with older age at diagnosis (HR, 1.08 [95% CI, 1.05-1.12] and 1.10 [95% CI, 1.06-1.14] per year of age, respectively; *P* < .001). Year of diagnosis and use of alkylating agents were not statistically significant for any of the cardiac end points.

**Table 2.** Subdistribution HRs for Grade 3 to 5 Cardiac Diseases From Fine and Gray Models Fitted to Hodgkin Lymphoma Survivors in the Childhood Cancer Survivor Study Cohort

Variable	Any cardiac disease		Coronary artery disease		Heart failure	
	Subdistribution HR (95% CI)	*P* value	Subdistribution HR (95% CI)	*P* value	Subdistribution HR (95% CI)	*P* value
Male (reference: female)	1.04 (0.84-1.30)	.72	1.72 (1.32-2.26)	<.001	0.49 (0.34-0.69)	<.001
Age at diagnosis, y	1.08 (1.05-1.12)	<.001	1.10 (1.06-1.14)	<.001	1.04 (0.99-1.09)	.12
Year of diagnosis 1995-1999 (reference: 1970-1974)	0.73 (0.37-1.43)	.35	0.57 (0.20-1.68)	.31	0.76 (0.33-1.78)	.53
Alkylating agent use (reference: no)	0.86 (0.68-1.07)	.18	0.82 (0.62-1.08)	.15	1.11 (0.78-1.58)	.57
Cumulative anthracycline dose, 100 mg/m^2^	1.16 (1.04-1.28)	.006	1.06 (0.91-1.22)	.48	1.49 (1.31-1.69)	<.001
Mean cardiac radiation therapy dose, 10 Gy	1.40 (1.26-1.55)	<.001	1.43 (1.26-1.62)	<.001	1.59 (1.36-1.86)	<.001

Abbreviation: HR, hazard ratio.

**Figure 1.**
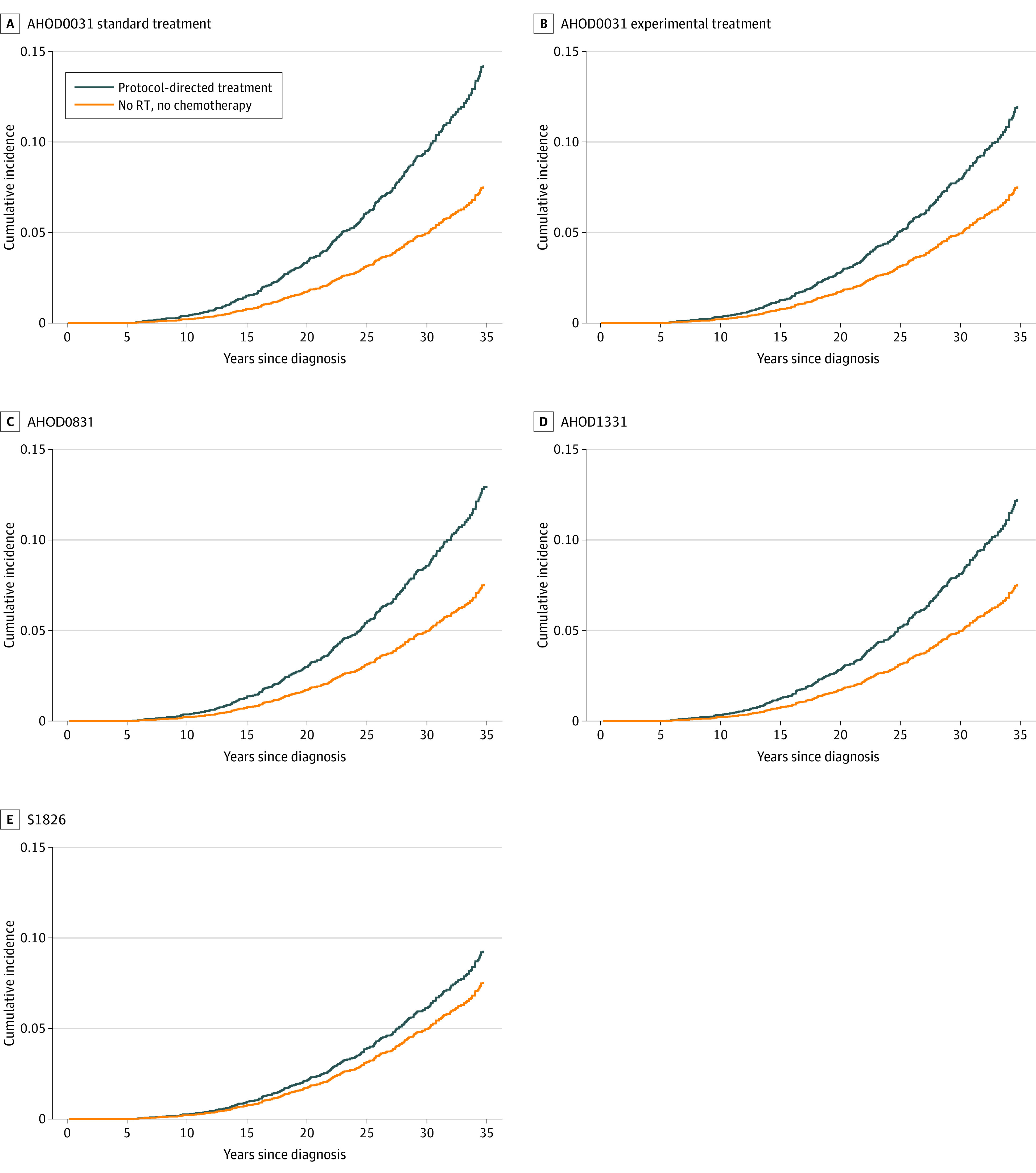
Estimated Cumulative Incidence of Grade 3 to 5 Cardiac Disease in 4 Children’s Oncology Group Hodgkin Lymphoma Trials A, AHOD0031 trial standard treatment (85% mediastinal radiation therapy [RT]); 30-year cumulative incidence (CI), 9.6%. B, Experimental treatment (40% mediastinal RT); 30-year CI, 8.0%. C, AHOD0831 trial; 30-year CI, 8.6%. D, AHOD1331 trial; 30-year CI, 8.2%. E, S1826 trial; 30-year CI, 6.2%. Results are shown for age 15 years at treatment, with equal proportions of males and females.

When these dose-risk associations were applied to patients enrolled in the COG trials, the estimated 30-year cumulative incidence of any grade 3 to 5 cardiac disease among 15-year-old patients (with equal proportions of males and females) in the AHOD0031 trial (enrolled 2002-2009) was estimated to be 9.6% (95% CI, 4.2%-16.4%) in the standard treatment group and 8.0% (95% CI, 3.6%-13.8%) in the experimental treatment group (Figure 1). In the subsequent AHOD0831 trial (enrolled 2009-2012), the estimated 30-year cumulative incidence of any cardiac disease was 8.6% (95% CI, 3.8%-14.9%); this was estimated to decrease with the successive trials to 8.2% (95% CI, 3.6%-14.3%) for AHOD1331 (enrolled 2015-2019) and 6.2% (95% CI, 2.7%-10.9%) for S1826 (enrolled 2019-2022). The expected rate in an untreated population was 5.0% (95% CI, 2.1%-9.3%). For most treatment exposures, the estimated incidences of CAD and HF were roughly equivalent (eTable 1 in Supplement 1). The estimated cumulative incidence of adverse cardiac outcomes associated with specific combinations of cardiac radiation dose and anthracycline exposure, treatment age, and sex are shown in eTables 1 to 15 and eFigure 1 in Supplement 1.

**Figure 2.**
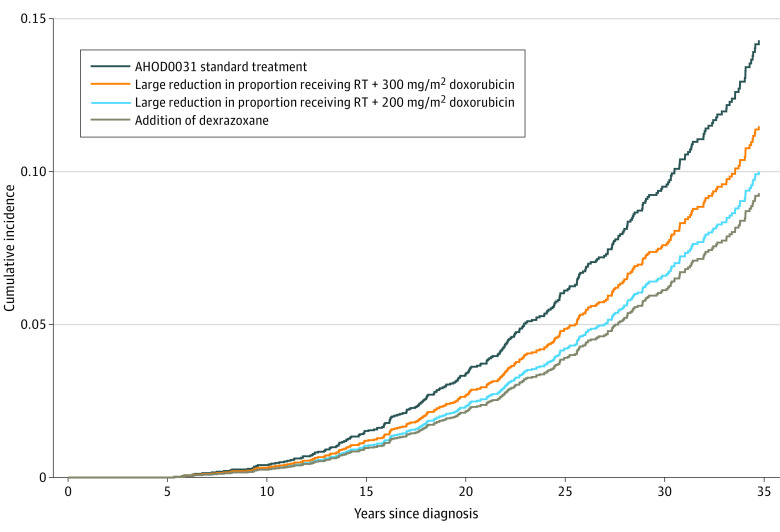
Association of Changing Individual Treatment Components on Cumulative Incidence of Cardiac Disease in the AHOD0031 Trial Treatment components comprised the following: AHOD0031 standard treatment (cumulative doxorubicin, 200 mg/m^2^; with 85% receiving mediastinal RT at a mean heart dose of 13.2 Gy and no dexrazoxane); reduction in RT use (cumulative doxorubicin, 200 mg/m^2^; with 1% receiving mediastinal RT at a mean heart dose of 4 Gy and no dexrazoxane); increase in cumulative doxorubicin dose (cumulative doxorubicin, 300 mg/m^2^; with 1% receiving mediastinal RT at a mean heart dose of 4 Gy and no dexrazoxane); and increase in dexrazoxane use (cumulative doxorubicin, 300 mg/m^2^; with 1% receiving mediastinal RT at a mean heart dose of 4 Gy and 80% receiving dexrazoxane). Results are shown for age 15 years at treatment, with an equal proportion of males and females. RT indicates radiation therapy.

Figure 2 illustrates the stepwise contributions of different treatment changes to the overall risk of late cardiac toxic effects as protocols evolved from the AHOD0031 trial through the S1826 trial. Starting with the AHOD0031 standard treatment group (200 mg/m^2^ doxorubicin; 85% receiving mediastinal RT, with a mean [SD] mean heart dose of 13.2 [5.7] Gy), the 30-year cumulative incidence of grade 3 to 5 cardiac disease was estimated to be 9.6% (95% CI, 4.2%-16.4%). Had the doxorubicin dose and dexrazoxane use remained constant, the reduction in the proportion of patients receiving mediastinal RT in the S1826 trial (to 0.4%) would reduce the 30-year incidence of grade 3 to 5 cardiac disease to 6.6% (95% CI, 2.9%-11.7%). However, with this RT use rate, the increase in the cumulative doxorubicin dose from 200 to 300 mg/m^2^ was estimated to increase this 30-year rate to 7.6% (95% CI, 3.3%-13.4%). Finally, given both of these changes in treatment exposure, the increased use of dexrazoxane cardioprotection to 80% of patients in the S1826 trial was expected to decrease the 30-year incidence of any cardiac toxic effects to 6.2% (95% CI, 2.7%-10.9%).

Based on the most recently published COG follow-up guidelines,^15^ 100% of survivors treated in all 4 HL trials evaluated would be recommended to undergo echocardiographic screening every 2 or 5 years following treatment. However, despite the decrease in RT use and lower estimated risk of cardiac morbidity, these findings suggest that the increase in the standard doxorubicin dose from 200 to 300 mg/m^2^ for all patients with high-risk HL will lead to an increase in the proportion of patients for whom 2-yearly echocardiography is recommended. For childhood survivors in the most recent S1826 trial, 100% are recommended to undergo 2-yearly echocardiography, whereas this proportion had been 85% for the AHOD0031 standard treatment group and as low as 40% for survivors in the AHOD0031 experimental treatment group and 58% for survivors treated in the AHOD0831 trial.

**Figure 3.**
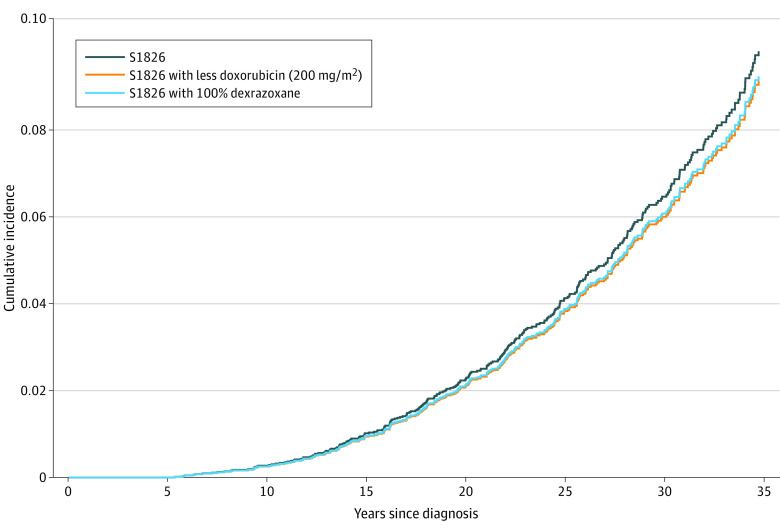
Estimated Outcomes of Modifying the S1826 Protocol to Further Reduce Cardiac Late Effects Thirty-year cumulative incidences of 6.2% (95% CI, 2.7%-10.9%) for the S1826 trial standard treatment, 5.7% (95% CI, 2.5%-10.3%) with the cumulative doxorubicin dose reduced to 200 mg/m^2^, and 5.8% (95% CI, 2.6%-10.5%) with dexrazoxane in all patients.

Figure 3 displays the estimated outcomes of potential future treatment modifications on cardiac risk moving forward from the S1826 protocol. Decreasing the cumulative doxorubicin dose from 300 to 200 mg/m^2^ or increasing dexrazoxane use from 80% to 100% was estimated to produce the greatest reductions in cardiac toxic effects, although the magnitude of reduction from either modification was small.

## Discussion

To our knowledge, this is the first study to provide quantitative estimates of potential consequences of individual HL protocol changes on late cardiac toxic effects, including estimates of the expected morbidity associated with modern trial protocols. Substantial efforts have been directed at decreasing late toxic effects from RT by limiting its use and improving RT techniques. However, the benefit of reducing treatment intensity must be weighed against the potential to increase the rate of relapse. Therefore, although RT was substantially reduced and refined, anthracycline doses were increased when analysis of the AHOD0031 trial suggested that 4 cycles of ABVE-PC (ie, 200 mg/m^2^) produced inadequate event-free survival in a subset of patients with high-risk HL.^8^ Subsequently, progression-free survival rates among children with advanced-stage HL treated in the AHOD1331 and S1826 trials are among the best reported in multi-institutional randomized trials,^10,16^ although the role of treatment changes in expected late effect risks has not previously been quantified. Our results suggest that for survivors treated in the most recent high-risk HL trial (S1826), the 30-year risk of serious cardiac morbidity will be increased less than 2% above the expected rate in an untreated population. Reassuringly, our results suggest that cardiac toxic effects are estimated to decrease from older to newer trials even in 12-year-old females who are most susceptible to anthracycline-induced HF (eFigure 2 in Supplement 1).^17^

Although considerable attention has been paid to limiting cardiotoxic exposures, the increasing use of dexrazoxane cardioprotection also plays an important role in reducing late cardiac morbidity among childhood HL survivors. Dexrazoxane, a topoisomerase II inhibitor that chelates iron and reduces reactive oxygen species formation, appears to protect the myocardium from anthracycline-induced cardiac toxic effects.^18^ A meta-analysis found that dexrazoxane was associated with a statistically significant reduction in clinical cardiac toxic effects among 8 nonrandomized studies (relative risk, 0.29), although each with less than 10 years of median follow-up. Chow et al^19,20^ examined long-term outcomes of dexrazoxane among 1308 survivors of childhood cancer treated in trials involving anthracycline, with a median follow-up of nearly 20 years. Randomization to dexrazoxane (vs no dexrazoxane) was associated with a significant decrease in serious cardiovascular outcomes (5.6% vs 17.6%; *P* = .02).^19^ The use of dexrazoxane has increased steadily over time, to approximately 80% of children in the most recent S1826 trial; our results suggest that further increasing dexrazoxane use to all patients may lead to a reduction in cardiac morbidity that would be roughly equivalent to decreasing the cumulative doxorubicin dose from 300 to 200 mg/m^2^. Notably, only 13% of patients aged older than 18 years were treated with cardioprotection in the AHOD1331 trial, suggesting that increasing dexrazoxane use in these older patients could provide a relatively simple means of reducing cardiac morbidity without the potential risk associated with reducing treatment intensity. However, among the components of treatment influencing cardiac risk, dexrazoxane is the least well studied, indicating an opportunity to better quantify its role in late cardiac toxic effects, including among young adults.

Another approach to limiting the late effects of therapy among childhood cancer survivors is to improve their long-term follow-up care. Guidelines recommending echocardiographic surveillance of survivors treated with cardiotoxic agents have been in place since 2003, with the frequency of testing dictated by the use of mediastinal RT and the dose of anthracyclines received. Our results reveal a paradox that despite a decrease in the expected rate of late cardiac morbidity associated with the most recent protocols, the frequency of COG guideline-recommended echocardiography is higher for these patients. For example, due to the increase in the proportion of survivors recommended to have 2-yearly echocardiography, guideline-compliant survivors treated in the S1826 trial would have roughly 33% more echocardiograms done over the course of a 30-year follow-up interval than survivors in the AHOD0831 trial. Overmedicalization with unnecessary tests may have negative consequences on quality of life among HL survivors by increasing anxiety and time away from work or school. Thus, in addition to continuing to modify protocols to further reduce cardiotoxic exposures, our findings suggest the need to continue the work done to refine cardiac follow-up guidelines to better align with the risks associated with treatment.

The face validity of our modeled cardiac toxic effect rates is supported by previously published studies on large pediatric cancer cohorts. In the German-Austrian Hodgkin lymphoma trials, the 25-year cumulative incidence of cardiac disease was 5% in patients treated with 20 Gy of mediastinal RT and 160 mg/m^2^ of doxorubicin, similar to our 25-year estimated cumulative incidence of cardiac disease of 6% in the AHOD0031 standard treatment group (prescribed a RT dose of 21 Gy and cumulative doxorubicin dose of 200 mg/m^2^).^21^ Similarly, in the Euro2K study, which comprised survivors of all types of pediatric cancers, the 25-year cumulative incidence of cardiac disease was approximately 6% for those receiving an anthracycline and a median heart dose of 0.1 to 14.9 Gy.^22^

### Limitations

This study has some limitations. The exploratory nature of applying the CCSS model to COG patients needs to be acknowledged, as differences between the 2 populations may confound cardiac risk estimates. For example, family history is an important component of inherited cardiac risk that we could not account for due to lack of data but would be valuable to integrate in future studies. Another limitation is that the cardiac outcomes in the CCSS were restricted to grade 3 to 5 events and were self-reported. Furthermore, mean heart dose is a robust but not completely comprehensive measure of radiation-induced cardiac risk, as it does not account for dose homogeneity or dose to cardiac substructures. Finally, immune checkpoint inhibitors have been associated with uncommon episodes of acute cardiac toxic effects. These episodes generally appear to be reversible but as these agents gain increasing use in HL management, their role in late cardiac outcomes must be determined.^23^

## Conclusions

This cohort study suggests that as HL trials have evolved, reductions in the proportion of patients receiving mediastinal RT, smaller RT target volumes, and increased use of dexrazoxane are estimated to offset the increase in doxorubicin dose and produce a net reduction in late cardiac disease. Additional studies on dexrazoxane are warranted to confirm whether its role in reducing cardiac toxic effects is maintained long term.

## Notes

Supplement 1. **eTable 1.** Thirty-Year Cumulative Incidence and 95% CIs (%) of Any Cardiac Disease, Coronary Artery Disease, and Heart Failure for Average 15-Year-Old Patient With Dexrazoxane According to Cumulative Anthracycline Dose and Mean Cardiac Radiotherapy Dose **eTable 2.** Thirty-Year Cumulative Incidence and 95% CIs (%) of Any Cardiac Disease, Coronary Artery Disease, and Heart Failure for Average 12-Year-Old Patient With Dexrazoxane According to Cumulative Anthracycline Dose and Mean Cardiac Radiotherapy Dose **eTable 3.** Thirty-Year Cumulative Incidence and 95% CIs (%) of Any Cardiac Disease, Coronary Artery Disease, and Heart Failure for Average 18-Year-Old Patient With Dexrazoxane According to Cumulative Anthracycline Dose and Mean Cardiac Radiotherapy Dose **eTable 4.** Thirty-Year Cumulative Incidence and 95% CIs (%) of Any Cardiac Disease, Coronary Artery Disease, and Heart Failure for Average 12-Year-Old Female With Dexrazoxane According to Cumulative Anthracycline Dose and Mean Cardiac Radiotherapy Dose **eTable 5.** Thirty-Year Cumulative Incidence and 95% CIs (%) of Any Cardiac Disease, Coronary Artery Disease, and Heart Failure for Average 12-Year-Old-Male With Dexrazoxane According to Cumulative Anthracycline Dose and Mean Cardiac Radiotherapy Dose **eTable 6.** Thirty-Year Cumulative Incidence and 95% CIs (%) of Any Cardiac Disease, Coronary Artery Disease and Heart Failure for Average 15-Year-Old Female With Dexrazoxane According to Cumulative Anthracycline Dose and Mean Cardiac Radiotherapy Dose **eTable 7.** Thirty-Year Cumulative Incidence and 95% CIs (%) of Any Cardiac Disease, Coronary Artery Disease, and Heart Failure for Average 15-Year-Old Male With Dexrazoxane According to Cumulative Anthracycline Dose and Mean Cardiac Radiotherapy Dose **eTable 8.** Thirty-Year Cumulative Incidence and 95% CIs (%) of Any Cardiac Disease, Coronary Artery Disease, and Heart Failure for Average 18-Year-Old Female With Dexrazoxane According to Cumulative Anthracycline Dose and Mean Cardiac Radiotherapy Dose **eTable 9.** Thirty-Year Cumulative Incidence and 95% CIs (%) of Any Cardiac Disease, Coronary Artery Disease, and Heart Failure for Average 18-Year-Old Male With Dexrazoxane According to Cumulative Anthracycline Dose and Mean Cardiac Radiotherapy Dose **eTable 10.** Thirty-Year Cumulative Incidence and 95% CIs (%) of Any Cardiac Disease, Coronary Artery Disease, and Heart Failure for Average 15-Year-Old White Female With Dexrazoxane According to Cumulative Anthracycline Dose and Mean Cardiac Radiotherapy Dose **eTable 11.** Thirty-Year Cumulative Incidence and 95% CIs (%) of Any Cardiac Disease, Coronary Artery Disease, and Heart Failure for Average 15-Year-Old Black Female With Dexrazoxane According to Cumulative Anthracycline Dose and Mean Cardiac Radiotherapy Dose **eTable 12.** Thirty-Year Cumulative Incidence and 95% CIs (%) of Any Cardiac Disease, Coronary Artery Disease, and Heart Failure for Average 15-Year-Old Hispanic Female With Dexrazoxane According to Cumulative Anthracycline Dose and Mean Cardiac Radiotherapy Dose **eTable 13.** Thirty-Year Cumulative Incidence and 95% CIs (%) of Any Cardiac Disease, Coronary Artery Disease, and Heart Failure for Average 15-Year-Old White Male With Dexrazoxane According to Cumulative Anthracycline Dose and Mean Cardiac Radiotherapy Dose **eTable 14.** Thirty-Year Cumulative Incidence and 95% CIs (%) of Any Cardiac Disease, Coronary Artery Disease, and Heart Failure for Average 15-Year-Old Black Male With Dexrazoxane According to Cumulative Anthracycline Dose and Mean Cardiac Radiotherapy Dose **eTable 15.** Thirty-Year Cumulative Incidence and 95% CIs (%) of Any Cardiac Disease, Coronary Artery Disease, and Heart Failure for Average 15-Year-Old Hispanic Male With Dexrazoxane According to Cumulative Anthracycline Dose and Mean Cardiac Radiotherapy Dose **eFigure 1.** Cumulative Incidence of Any Cardiac Disease Based on Mean Heart Dose for Average 15-Year-Old Patient **eFigure 2.** Thirty-Year Cumulative Incidence of Any Cardiac Disease in the Average 12-Year-Old Female on Each Trial

Supplement 2.
Data Sharing Statement
