# Supplementary material for: Late Cardiac Toxic Effects Associated With Treatment Protocols for Hodgkin Lymphoma in Children
Source: JAMA Netw Open. 2024 Jan 19;7(1):e2351062. doi: 10.1001/jamanetworkopen.2023.51062 (PMC10799264; doi:10.1001/jamanetworkopen.2023.51062)
Supplement: Supplement 1. — eTable 1. Thirty-Year Cumulative Incidence and 95% CIs (%) of Any Cardiac Disease, Coronary Artery Disease, and Heart Failure for Average 15-Year-Old Patient With Dexrazoxane According to Cumulative Anthracycline Dose and Mean Cardiac Radiotherapy Dose eTable 2. Thirty-Year Cumulative Incidence and 95% CIs (%) of Any Cardiac Disease, Coronary Artery Disease, and Heart Failure for Average 12-Year-Old Patient With Dexrazoxane According to Cumulative Anthracycline Dose and Mean Cardiac Radiotherapy Dose eTable 3. Thirty-Year Cumulative Incidence and 95% CIs (%) of Any Cardiac Disease, Coronary Artery Disease, and Heart Failure for Average 18-Year-Old Patient With Dexrazoxane According to Cumulative Anthracycline Dose and Mean Cardiac Radiotherapy Dose eTable 4. Thirty-Year Cumulative Incidence and 95% CIs (%) of Any Cardiac Disease, Coronary Artery Disease, and Heart Failure for Average 12-Year-Old Female With Dexrazoxane According to Cumulative Anthracycline Dose and Mean Cardiac Radiotherapy Dose eTable 5. Thirty-Year Cumulative Incidence and 95% CIs (%) of Any Cardiac Disease, Coronary Artery Disease, and Heart Failure for Average 12-Year-Old-Male With Dexrazoxane According to Cumulative Anthracycline Dose and Mean Cardiac Radiotherapy Dose eTable 6. Thirty-Year Cumulative Incidence and 95% CIs (%) of Any Cardiac Disease, Coronary Artery Disease and Heart Failure for Average 15-Year-Old Female With Dexrazoxane According to Cumulative Anthracycline Dose and Mean Cardiac Radiotherapy Dose eTable 7. Thirty-Year Cumulative Incidence and 95% CIs (%) of Any Cardiac Disease, Coronary Artery Disease, and Heart Failure for Average 15-Year-Old Male With Dexrazoxane According to Cumulative Anthracycline Dose and Mean Cardiac Radiotherapy Dose eTable 8. Thirty-Year Cumulative Incidence and 95% CIs (%) of Any Cardiac Disease, Coronary Artery Disease, and Heart Failure for Average 18-Year-Old Female With Dexrazoxane According to Cumulative Anthracycline Dose and Mean Cardiac Radi [file jamanetwopen-e2351062-s001.pdf]

Supplementary Online Content

Lo AC, Liu A, Liu Q, et al. Late cardiac toxic effects associated with treatment protocols for Hodgkin lymphoma in children. *JAMA Netw Open*. 2024;7(1):e2351062. doi:10.1001/jamanetworkopen.2023.51062

eTable 1. Thirty-Year Cumulative Incidence and 95% CIs (%) of Any Cardiac Disease, Coronary Artery Disease, and Heart Failure for Average 15-Year-Old Patient With Dexrazoxane According to Cumulative Anthracycline Dose and Mean Cardiac Radiotherapy Dose

eTable 2. Thirty-Year Cumulative Incidence and 95% CIs (%) of Any Cardiac Disease, Coronary Artery Disease, and Heart Failure for Average 12-Year-Old Patient With Dexrazoxane According to Cumulative Anthracycline Dose and Mean Cardiac Radiotherapy Dose

eTable 3. Thirty-Year Cumulative Incidence and 95% CIs (%) of Any Cardiac Disease, Coronary Artery Disease, and Heart Failure for Average 18-Year-Old Patient With Dexrazoxane According to Cumulative Anthracycline Dose and Mean Cardiac Radiotherapy Dose

eTable 4. Thirty-Year Cumulative Incidence and 95% CIs (%) of Any Cardiac Disease, Coronary Artery Disease, and Heart Failure for Average 12-Year-Old Female With Dexrazoxane According to Cumulative Anthracycline Dose and Mean Cardiac Radiotherapy Dose

eTable 5. Thirty-Year Cumulative Incidence and 95% CIs (%) of Any Cardiac Disease, Coronary Artery Disease, and Heart Failure for Average 12-Year-Old-Male With Dexrazoxane According to Cumulative Anthracycline Dose and Mean Cardiac Radiotherapy Dose

eTable 6. Thirty-Year Cumulative Incidence and 95% CIs (%) of Any Cardiac Disease, Coronary Artery Disease and Heart Failure for Average 15-Year-Old Female With Dexrazoxane According to Cumulative Anthracycline Dose and Mean Cardiac Radiotherapy Dose

eTable 7. Thirty-Year Cumulative Incidence and 95% CIs (%) of Any Cardiac Disease, Coronary Artery Disease, and Heart Failure for Average 15-Year-Old Male With Dexrazoxane According to Cumulative Anthracycline Dose and Mean Cardiac Radiotherapy Dose

eTable 8. Thirty-Year Cumulative Incidence and 95% CIs (%) of Any Cardiac Disease, Coronary Artery Disease, and Heart Failure for Average 18-Year-Old Female With Dexrazoxane According to Cumulative Anthracycline Dose and Mean Cardiac Radiotherapy Dose

© 2024 Lo AC et al. *JAMA Network Open.*

eTable 9. Thirty-Year Cumulative Incidence and 95% CIs (%) of Any Cardiac Disease, Coronary Artery Disease, and Heart Failure for Average 18-Year-Old Male With Dexrazoxane According to Cumulative Anthracycline Dose and Mean Cardiac Radiotherapy Dose

eTable 10. Thirty-Year Cumulative Incidence and 95% CIs (%) of Any Cardiac Disease, Coronary Artery Disease, and Heart Failure for Average 15-Year-Old White Female With Dexrazoxane According to Cumulative Anthracycline Dose and Mean Cardiac Radiotherapy Dose

eTable 11. Thirty-Year Cumulative Incidence and 95% CIs (%) of Any Cardiac Disease, Coronary Artery Disease, and Heart Failure for Average 15-Year-Old Black Female With Dexrazoxane According to Cumulative Anthracycline Dose and Mean Cardiac Radiotherapy Dose

eTable 12. Thirty-Year Cumulative Incidence and 95% CIs (%) of Any Cardiac Disease, Coronary Artery Disease, and Heart Failure for Average 15-Year-Old Hispanic Female With Dexrazoxane According to Cumulative Anthracycline Dose and Mean Cardiac Radiotherapy Dose

eTable 13. Thirty-Year Cumulative Incidence and 95% CIs (%) of Any Cardiac Disease, Coronary Artery Disease, and Heart Failure for Average 15-Year-Old White Male With Dexrazoxane According to Cumulative Anthracycline Dose and Mean Cardiac Radiotherapy Dose

eTable 14. Thirty-Year Cumulative Incidence and 95% CIs (%) of Any Cardiac Disease, Coronary Artery Disease, and Heart Failure for Average 15-Year-Old Black Male With Dexrazoxane According to Cumulative Anthracycline Dose and Mean Cardiac Radiotherapy Dose

eTable 15. Thirty-Year Cumulative Incidence and 95% CIs (%) of Any Cardiac Disease, Coronary Artery Disease, and Heart Failure for Average 15-Year-Old Hispanic Male With Dexrazoxane According to Cumulative Anthracycline Dose and Mean Cardiac Radiotherapy Dose

eFigure 1. Cumulative Incidence of Any Cardiac Disease Based on Mean Heart Dose for Average 15-Year-Old Patient

eFigure 2. Thirty-Year Cumulative Incidence of Any Cardiac Disease in the Average 12-Year-Old Female on Each Trial

This supplementary material has been provided by the authors to give readers additional information about their work.

© 2024 Lo AC et al. *JAMA Network Open.*

eTable 1. Thirty-Year Cumulative Incidence and 95% CIs (%) of Any Cardiac Disease, Coronary Artery Disease, and Heart Failure for Average 15-Year-Old Patient With Dexrazoxane According to Cumulative Anthracycline Dose and Mean Cardiac Radiotherapy Dose

	Cumulative Anthracycline Dose (mg/m2)								
	Any Cardiac Disease			Coronary Artery Disease			Heart Failure		
Mean cardiac RT dose, Gy	200 mg/m2	250 mg/m2	300 mg/m2	200 mg/m2	250 mg/m2	300 mg/m2	200 mg/m2	250 mg/m2	300 mg/m2
0	5.4 (2.4, 10)	5.5 (2.5, 10.1)	5.7 (2.6, 10.5)	2.7 (0.6, 6.5)	2.7 (0.6, 6.5)	2.8 (0.6, 6.5)	2.2 (0.7, 4.7)	2.5 (0.7, 5.2)	2.8 (0.8, 5.8)
5	6.3 (2.8, 11.7)	6.5 (2.9, 11.9)	6.7 (3.0, 12.2)	3.2 (0.7, 7.6)	3.3 (0.7, 7.6)	3.3 (0.7, 7.6)	2.8 (0.8, 5.8)	3.1 (0.9, 6.4)	3.5 (1.1, 7.0)
10	7.4 (3.4, 0.136)	7.6 (3.5, 14.0)	7.8 (3.6, 14.3)	3.9 (0.8, 8.9)	3.9 (0.9, 9.0)	4.0 (0.9, 9.0)	3.5 (1.1, 7.2)	3.9 (1.2, 7.9)	4.4 (1.4, 8.7)
15	8.7 (4.1, 15.8)	8.9 (4.2, 16.2)	9.2 (4.2, 16.6)	4.6 (1.0, 10.8)	4.7 (1.0, 10.7)	4.7 (1.0, 10.8)	4.4 (1.4, 9.1)	4.9 (1.6, 9.8)	5.5 (1.8, 10.8)
20	10.2 (4.8, 18.6)	10.4 (4.9, 18.9)	10.8 (5.1, 19.3)	5.5 (1.2, 12.9)	5.5 (1.2, 13.0)	5.6 (1.2, 13.0)	5.6 (1.8, 11.3)	6.2 (2.1, 12.3)	6.9 (2.3, 13.4)

© 2024 Lo AC et al. *JAMA Network Open.*

eTable 2. Thirty-Year Cumulative Incidence and 95% CIs (%) of Any Cardiac Disease, Coronary Artery Disease, and Heart Failure for Average 12-Year-Old Patient With Dexrazoxane According to Cumulative Anthracycline Dose and Mean Cardiac Radiotherapy Dose

12 year old	Cumulative Anthracycline Dose (mg/m2)								
	Any Cardiac Disease			Coronary Artery Disease			Heart Failure		
Mean cardiac RT dose, Gy	200 mg/m2	250 mg/m2	300 mg/m2	200 mg/m2	250 mg/m2	300 mg/m2	200 mg/m2	250 mg/m2	300 mg/m2
0	4.2 (1.9, 8.0)	4.3 (2.0, 8.3)	4.5 (2.0, 8.4)	2.1 (0.4, 4.9)	2.1 (0.4, 5.0)	2.1 (0.4, 5.0)	2.0 (0.6, 4.3)	2.2 (0.7, 4.7)	2.5 (0.8, 5.2)
5	5.0 (2.3, 9.2)	5.1 (2.3, 9.4)	5.3 (2.4, 9.7)	2.5 (0.5, 6.0)	2.5 (0.5, 6.0)	2.5 (0.5, 6.0)	2.5 (0.8, 5.3)	2.8 (0.9, 5.8)	3.2 (0.9, 6.4)
10	5.9 (2.7, 10.9)	6.0 (2.7, 11.1)	6.1 (2.8, 11.3)	3.0 (0.6, 7.2)	3.0 (0.6, 7.2)	3.0 (0.6, 7.2)	3.1 (1.0, 6.5)	3.5 (1.1, 7.2)	3.9 (1.2, 7.9)
15	6.9 (3.1, 12.8)	7.1 (3.2, 13.1)	7.3 (3.3, 13.4)	3.5 (0.8, 8.6)	3.6 (0.8, 8.6)	3.6 (0.8, 8.6)	3.9 (1.3, 8.1)	4.4 (1.4, 8.9)	4.9 (1.6, 9.8)
20	8.1 (3.6, 15.0)	8.3 (3.8, 15.4)	8.6 (3.9, 15.7)	4.2 (0.9, 10.3)	4.2 (0.9, 10.3)	4.3 (0.9, 10.3)	4.9 (1.7, 10.1)	5.5 (1.8, 11.0)	6.0 (2.0, 11.9)

eTable 3. Thirty-Year Cumulative Incidence and 95% CIs (%) of Any Cardiac Disease, Coronary Artery Disease, and Heart Failure for Average 18-Year-Old Patient With Dexrazoxane According to Cumulative Anthracycline Dose and Mean Cardiac Radiotherapy Dose

18 year old	Cumulative Anthracycline Dose (mg/m2)								
	Any Cardiac Disease			Coronary Artery Disease			Heart Failure		
Mean cardiac RT dose, Gy	200 mg/m2	250 mg/m2	300 mg/m2	200 mg/m2	250 mg/m2	300 mg/m2	200 mg/m2	250 mg/m2	300 mg/m2
0	6.9 (3.0, 12.9)	7.0 (3.1, 13.2)	7.2 (3.2, 13.4)	3.5 (0.8, 8.6)	3.6 (0.8, 8.5)	3.7 (0.8, 8.5)	2.5 (0.7, 5.5)	2.8 (0.8, 6.1)	3.1 (0.9, 6.8)
5	8.0 (3.6, 14.8)	8.2 (3.7, 15.1)	8.5 (3.7, 15.5)	4.3 (0.9, 10.0)	4.3 (1.0, 10.0)	4.4 (1.0, 10.1)	3.2 (0.9, 6.8)	3.5 (1.1, 7.4)	3.9 (1.2, 8.2)
10	9.4 (4.2, 17.1)	9.6 (4.4, 17.3)	9.9 (4.5, 17.7)	5.1 (1.1, 11.8)	5.1 (1.1, 11.9)	5.2 (1.1, 11.9)	4.0 (1.2, 8.3)	4.4 (1.4, 9.1)	4.9 (1.5, 10.1)
15	11.0 (5.1, 19.7)	11.3 (5.2, 20.1)	11.6 (5.4, 20.5)	6.1 (1.3, 14.0)	6.1 (1.3, 14.0)	6.2 (1.4, 14.0)	5.0 (1.6, 10.3)	5.5 (1.8, 11.2)	6.1 (2.0, 12.1)
20	12.9 (6.0, 23.2)	13.2 (6.2, 23.5)	13.6 (6.4, 23.8)	7.2 (1.6, 16.7)	7.2 (1.6, 16.9)	7.3 (1.6, 16.8)	6.2 (2.1, 12.6)	6.9 (2.3, 13.7)	7.6 (2.5, 15.0)

© 2024 Lo AC et al. *JAMA Network Open.*

eTable 4. Thirty-Year Cumulative Incidence and 95% CIs (%) of Any Cardiac Disease, Coronary Artery Disease, and Heart Failure for Average 12-Year-Old Female With Dexrazoxane According to Cumulative Anthracycline Dose and Mean Cardiac Radiotherapy Dose

12 year old female	Cumulative Anthracycline Dose (mg/m2)								
	Any Cardiac Disease			Coronary Artery Disease			Heart Failure		
Mean cardiac RT dose, Gy	200 mg/m2	250 mg/m2	300 mg/m2	200 mg/m2	250 mg/m2	300 mg/m2	200 mg/m2	250 mg/m2	300 mg/m2
0	4.1 (1.8, 7.8)	4.2 (1.9, 7.9)	4.4 (1.9, 8.1)	1.5 (0.3, 3.9)	1.5 (0.3, 3.9)	1.5 (0.3, 3.9)	2.6 (0.8, 5.9)	2.9 (0.9, 6.4)	3.3 (1.0, 7.0)
5	4.9 (2.1, 9.1)	5.0 (2.2, 9.4)	5.1 (2.3, 9.5)	1.8 (0.4, 4.5)	1.8 (0.4, 4.5)	1.8 (0.4, 4.4)	3.3 (1.0, 7.1)	3.7 (1.1, 7.8)	4.2 (1.3, 8.5)
10	5.7 (2.6, 10.7)	5.8 (2.6, 11.0)	6.0 (2.7, 11.3)	2.1 (0.4, 5.4)	2.2 (0.4, 5.4)	2.2 (0.4, 5.4)	4.2 (1.3, 8.9)	4.6 (1.5, 9.6)	5.2 (1.6, 10.4)
15	6.7 (3.0, 12.5)	6.9 (3.1, 12.8)	7.1 (3.2, 13.2)	2.6 (0.5, 6.5)	2.6 (0.5, 6.6)	2.6 (0.5, 6.6)	5.3 (1.8, 11.2)	5.8 (1.9, 12.0)	6.5 (2.1, 12.9)
20	7.9 (3.5, 14.6)	8.1 (3.6, 15.0)	8.3 (3.7, 15.3)	3.1 (0.6, 7.8)	3.1 (0.6, 7.8)	3.1 (0.6, 7.9)	6.6 (2.3, 13.6)	7.3 (2.5, 14.7)	8.1 (2.7, 15.9)

© 2024 Lo AC et al. *JAMA Network Open.*

eTable 5. Thirty-Year Cumulative Incidence and 95% Cis (%) of Any Cardiac Disease, Coronary Artery Disease, and Heart Failure for Average 12-Year-Old-Male With Dexrazoxane According to Cumulative Anthracycline Dose and Mean Cardiac Radiotherapy Dose

**12 year old male**	**Cumulative Anthracycline Dose (mg/m2)**								
	**Any Cardiac Disease**			**Coronary Artery Disease**			**Heart Failure**		
**Mean cardiac RT dose, Gy**	**200 mg/m2**	**250 mg/m2**	**300 mg/m2**	**200 mg/m2**	**250 mg/m2**	**300 mg/m2**	**200 mg/m2**	**250 mg/m2**	**300 mg/m2**
0	4.3 (2.0, 8.2)	4.4 (2.0, 8.5)	4.6 (2.1, 8.7)	2.6 (0.5, 6.3)	2.6 (0.5, 6.3)	2.7 (0.5, 6.4)	1.3 (0.4, 3.0)	1.4 (0.4, 3.3)	1.6 (0.5, 3.6)
5	5.1 (2.3, 9.6)	5.2 (2.4, 9.8)	5.4 (2.4, 10.0)	3.1 (0.7, 7.4)	3.2 (0.7, 7.3)	3.2 (0.7, 7.4)	1.6 (0.5, 3.7)	1.8 (0.6, 4.1)	2.0 (0.6, 4.4)
10	6.0 (2.7, 11.3)	6.1 (2.8, 11.6)	6.3 (2.9, 11.8)	3.7 (0.8, 9.0)	3.8 (0.8, 9.0)	3.8 (0.8, 9.0)	2.1 (0.7, 4.6)	2.3 (0.7, 5.0)	2.6 (0.8, 5.5)
15	7.0 (3.2, 13.2)	7.2 (3.2, 13.4)	7.4 (3.4, 13.7)	4.4 (0.9, 10.7)	4.4 (1.0, 10.6)	4.5 (1.0, 10.7)	2.6 (0.9, 5.8)	2.9 (0.9, 6.3)	3.2 (1.1, 6.8)
20	8.2 (3.7, 15.5)	8.5 (3.9, 15.6)	8.7 (4.0, 16.0)	5.3 (1.1, 12.7)	5.3 (1.1, 12.8)	5.4 (1.1, 12.8)	3.2 (1.1, 7.3)	3.6 (1.2, 7.9)	4.0 (1.3, 8.5)

© 2024 Lo AC et al. *JAMA Network Open.*

eTable 6. Thirty-Year Cumulative Incidence and 95% CIs (%) of Any Cardiac Disease, Coronary Artery Disease and Heart Failure for Average 15-Year-Old Female With Dexrazoxane According to Cumulative Anthracycline Dose and Mean Cardiac Radiotherapy Dose

**15 year old females**	**Cumulative Anthracycline Dose (mg/m2)**								
	**Any Cardiac Disease**			**Coronary Artery Disease**			**Heart Failure**		
**Mean cardiac RT dose, Gy**	**200 mg/m2**	**250 mg/m2**	**300 mg/m2**	**200 mg/m2**	**250 mg/m2**	**300 mg/m2**	**200 mg/m2**	**250 mg/m2**	**300 mg/m2**
0	5.3 (2.3, 9.8)	5.4 (2.4, 9.9)	5.6 (2.4, 10.2)	2.0 (0.4, 4.9)	2.0 (0.4, 4.8)	2.0 (0.4, 4.9)	3.0 (0.9, 6.4)	3.3 (1.0, 7.0)	3.7 (1.1, 7.8)
5	6.2 (2.8, 11.5)	6.4 (2.9, 11.8)	6.5 (2.9, 12.0)	2.4 (0.5, 5.8)	2.4 (0.5, 5.8)	2.4 (0.5, 5.8)	3.7 (1.1, 7.8)	4.1 (1.3, 8.7)	4.6 (1.4, 9.5)
10	7.3 (3.3, 13.3)	7.5 (3.4, 13.6)	7.7 (3.5, 14.0)	2.9 (0.6, 6.9)	2.9 (0.6, 6.9)	2.9 (0.6, 7.0)	4.7 (1.5, 9.8)	5.2 (1.6, 10.6)	5.8 (1.8, 11.6)
15	8.4 (3.9, 15.6)	8.7 (4.0, 15.9)	8.9 (4.1, 16.3)	3.4 (0.7, 8.3)	3.5 (0.7, 8.3)	3.5 (0.7, 8.4)	5.9 (1.9, 12.1)	6.5 (2.1, 13.1)	7.2 (2.3, 14.3)
20	10.0 (4.5, 18.2)	10.2 (4.7, 18.6)	10.4 (4.8, 19.1)	4.1 (0.8, 10.0)	4.1 (0.8, 10.1)	4.2 (0.8, 10.1)	7.4 (2.5, 15.0)	8.1 (2.8, 16.2)	9.0 (3.1, 17.6)

© 2024 Lo AC et al. *JAMA Network Open.*

eTable 7. Thirty-Year Cumulative Incidence and 95% CIs (%) of Any Cardiac Disease, Coronary Artery Disease, and Heart Failure for Average 15-Year-Old Male With Dexrazoxane According to Cumulative Anthracycline Dose and Mean Cardiac Radiotherapy Dose

**15 year old male**	**Cumulative Anthracycline Dose (mg/m2)**								
	**Any Cardiac Disease**			**Coronary Artery Disease**			**Heart Failure**		
**Mean cardiac RT dose, Gy**	**200 mg/m2**	**250 mg/m2**	**300 mg/m2**	**200 mg/m2**	**250 mg/m2**	**300 mg/m2**	**200 mg/m2**	**250 mg/m2**	**300 mg/m2**
0	5.5 (2.5, 10.3)	5.6 (2.6, 10.5)	5.8 (2.7, 10.7)	3.4 (0.7, 8.2)	3.5 (0.7, 8.1)	3.5 (0.8, 8.2)	1.4 (0.4, 3.5)	1.6 (0.5, 3.8)	1.8 (0.5, 4.2)
5	6.5 (3.0, 12.0)	6.6 (3.1, 12.2)	6.8 (3.2, 12.6)	4.1 (0.9, 9.6)	4.2 (0.9, 9.6)	4.2 (0.9, 9.6)	1.8 (0.6, 4.2)	2.0 (0.6, 4.5)	2.3 (0.7, 5.0)
10	7.5 (3.5, 14.0)	7.8 (3.6, 14.2)	8.0 (3.7, 14.6)	4.9 (1.0, 11.3)	5.0 (1.0, 11.2)	5.0 (1.1, 11.4)	2.3 (0.7, 5.3)	2.6 (0.8, 5.7)	2.9 (0.9, 6.2)
15	8.9 (4.1, 16.3)	9.1 (4.3, 16.7)	9.4 (4.3, 17.0)	5.9 (1.2, 13.3)	5.9 (1.3, 13.2)	5.9 (1.3, 13.5)	2.9 (0.9, 6.5)	3.2 (1.0, 7.1)	3.6 (1.2, 7.8)
20	10.4 (4.8, 18.9)	10.7 (4.9, 19.3)	11.0 (5.1, 19.7)	6.9 (1.5, 15.9)	6.9 (1.5, 16.0)	7.0 (1.5, 16.1)	3.6 (1.2, 8.1)	4.1 (1.3, 8.8)	4.6 (1.5, 9.6)

© 2024 Lo AC et al. *JAMA Network Open.*

eTable 8. Thirty-Year Cumulative Incidence and 95% CIs (%) of Any Cardiac Disease, Coronary Artery Disease, and Heart Failure for Average 18-Year-Old Female With Dexrazoxane According to Cumulative Anthracycline Dose and Mean Cardiac Radiotherapy Dose

18 year old female	Cumulative Anthracycline Dose (mg/m2)								
	Any Cardiac Disease			Coronary Artery Disease			Heart Failure		
Mean cardiac RT dose, Gy	200 mg/m2	250 mg/m2	300 mg/m2	200 mg/m2	250 mg/m2	300 mg/m2	200 mg/m2	250 mg/m2	300 mg/m2
0	6.7 (2.8, 12.5)	6.9 (2.9, 12.7)	7.1 (3.0, 13.0)	2.6 (0.6, 6.4)	2.6 (0.6, 6.4)	2.7 (0.6, 6.5)	3.4 (0.9, 7.3)	3.7 (1.1, 8.1)	4.1 (1.2, 8.9)
5	7.9 (3.4, 14.4)	8.1 (3.5, 14.8)	8.3 (3.6, 15.1)	3.1 (0.7, 7.5)	3.2 (0.7, 7.6)	3.2 (0.7, 7.6)	4.2 (1.2, 8.9)	4.6 (1.4, 9.7)	5.2 (1.6, 10.7)
10	9.2 (4.1, 16.7)	9.4 (4.2, 17.0)	9.7 (4.4, 17.7)	3.8 (0.8, 9.1)	3.8 (0.8, 9.1)	3.8 (0.8, 9.1)	5.3 (1.6, 11.0)	5.8 (1.8, 11.9)	6.5 (2.0, 13.2)
15	10.7 (4.9, 19.4)	11.0 (5.0, 20.0)	11.3 (5.2, 20.4)	4.5 (0.9, 10.9)	4.5 (0.9, 10.8)	4.6 (1.0, 10.9)	6.6 (2.1, 13.5)	7.3 (2.3, 14.7)	8.1 (2.5, 16.2)
20	12.6 (5.7, 22.6)	12.9 (5.9, 23.1)	13.2 (6.1, 23.6)	5.4 (1.1, 12.8)	5.4 (1.1, 12.9)	5.5 (1.1, 13.1)	8.3 (2.7, 16.8)	9.1 (3.0, 18.0)	10.1 (3.3, 19.8)

© 2024 Lo AC et al. *JAMA Network Open.*

eTable 9. Thirty-Year Cumulative Incidence and 95% CIs (%) of Any Cardiac Disease, Coronary Artery Disease, and Heart Failure for Average 18-Year-Old Male With Dexrazoxane According to Cumulative Anthracycline Dose and Mean Cardiac Radiotherapy Dose

**18 year old male**	**Cumulative Anthracycline Dose (mg/m2)**								
	**Any Cardiac Disease**			**Coronary Artery Disease**			**Heart Failure**		
**Mean cardiac RT dose, Gy**	**200 mg/m2**	**250 mg/m2**	**300 mg/m2**	**200 mg/m2**	**250 mg/m2**	**300 mg/m2**	**200 mg/m2**	**250 mg/m2**	**300 mg/m2**
0	7.0 (3.1, 13.0)	7.2 (3.2, 13.1)	7.4 (3.3, 13.5)	4.5 (1.0, 10.8)	4.6 (1.0, 10.9)	4.6 (1.0, 10.9)	1.6 (0.5, 3.9)	1.8 (0.5, 4.3)	2.0 (0.6, 4.9)
5	8.2 (3.7, 15.0)	8.4 (3.9, 15.3)	8.7 (4.1, 15.6)	5.4 (1.1, 12.7)	5.5 (1.2, 12.7)	5.5 (1.2, 12.8)	2.1 (0.6, 4.8)	2.3 (0.7, 5.3)	2.6 (0.8, 5.9)
10	9.6 (4.4, 17.5)	9.9 (4.6, 17.8)	10.2 (4.7, 18.1)	6.5 (1.4, 15.1)	6.5 (1.4, 15.2)	6.6 (1.4, 15.1)	2.6 (0.8, 6.0)	2.9 (0.9, 6.6)	3.2 (1.0, 7.2)
15	11.3 (5.2, 20.2)	11.6 (5.3, 20.6)	11.9 (5.5, 21.1)	7.7 (1.7, 17.6)	7.8 (1.7, 17.7)	7.8 (1.7, 17.8)	3.3 (1.0, 7.4)	3.6 (1.1, 8.1)	4.1 (1.2, 8.9)
20	13.1 (6.3, 23.5)	13.5 (6.4, 23.9)	13.9 (6.6, 24.1)	9.1 (2.0, 20.8)	9.2 (2.0, 21.0)	9.3 (2.0, 21.0)	4.1 (1.3, 9.2)	4.6 (1.5, 10.0)	5.1 (1.6, 10.9)

© 2024 Lo AC et al. *JAMA Network Open.*

eTable 10. Thirty-Year Cumulative Incidence and 95% CIs (%) of Any Cardiac Disease, Coronary Artery Disease, and Heart Failure for Average 15-Year-Old White Female With Dexrazoxane According to Cumulative Anthracycline Dose and Mean Cardiac Radiotherapy Dose

15 year old white female	Cumulative Anthracycline Dose (mg/m2)								
	Any Cardiac Disease			Coronary Artery Disease			Heart Failure		
Mean cardiac RT dose, Gy	200 mg/m2	250 mg/m2	300 mg/m2	200 mg/m2	250 mg/m2	300 mg/m2	200 mg/m2	250 mg/m2	300 mg/m2
0	5.1 (2.2, 9.5)	5.3 (2.2, 9.6)	5.4 (2.3, 9.9)	1.9 (0.4, 4.9)	1.9 (0.4, 4.9)	2.0 (0.4, 5.0)	2.8 (0.8, 6.0)	3.1 (0.9, 6.6)	3.5 (1.0, 7.3)
5	6.0 (2.5, 11.0)	6.2 (2.6, 11.3)	6.3 (2.7, 11.6)	2.3 (0.5, 5.8)	2.3 (0.5, 5.8)	2.4 (0.5, 5.9)	3.5 (1.1, 7.3)	3.9 (1.2, 8.1)	4.4 (1.4, 8.9)
10	7.0 (3.1, 12.9)	7.2 (3.1, 13.4)	7.4 (3.2, 13.6)	2.8 (0.5, 6.9)	2.8 (0.5, 6.9)	2.8 (0.6, 6.9)	4.4 (1.4, 9.1)	4.9 (1.6, 9.9)	5.5 (1.8, 10.9)
15	8.2 (3.7, 15.1)	8.4 (3.7, 15.6)	8.7 (3.9, 16.0)	3.3 (0.7, 8.2)	3.3 (0.7, 8.2)	3.4 (0.7, 8.2)	5.6 (1.8, 11.3)	6.1 (2.1, 12.2)	6.9 (2.3, 13.4)
20	9.6 (4.2, 17.6)	9.8 (4.4, 18.1)	10.1 (4.6, 18.7)	4.0 (0.8, 9.8)	4.0 (0.8, 9.8)	4.0 (0.8, 9.9)	6.9 (2.4, 13.9)	7.6 (2.7, 15.1)	8.5 (3.0, 16.5)

© 2024 Lo AC et al. *JAMA Network Open.*

eTable 11. Thirty-Year Cumulative Incidence and 95% CIs (%) of Any Cardiac Disease, Coronary Artery Disease, and Heart Failure for Average 15-Year-Old Black Female With Dexrazoxane According to Cumulative Anthracycline Dose and Mean Cardiac Radiotherapy Dose

**15 year old black female**	**Cumulative Anthracycline Dose (mg/m2)**								
	**Any Cardiac Disease**			**Coronary Artery Disease**			**Heart Failure**		
**Mean cardiac RT dose, Gy**	**200 mg/m2**	**250 mg/m2**	**300 mg/m2**	**200 mg/m2**	**250 mg/m2**	**300 mg/m2**	**200 mg/m2**	**250 mg/m2**	**300 mg/m2**
0	6.2 (2.5, 12.1)	6.4 (2.5, 12.3)	6.6 (2.6, 12.5)	2.2 (0.4, 6.0)	2.2 (0.4, 6.0)	2.2 (0.4, 6.0)	3.8 (1.1, 8.7)	4.3 (1.2, 9.6)	4.8 (1.4, 10.7)
5	7.3 (3.0, 13.9)	7.4 (3.0, 14.2)	7.7 (3.1, 14.4)	2.6 (0.5, 7.1)	2.6 (0.5, 7.1)	2.7 (0.5, 7.1)	4.8 (1.4, 10.6)	5.3 (1.6, 11.5)	5.9 (1.8, 12.8)
10	8.5 (3.6, 16.0)	8.7 (3.7, 16.3)	9.0 (3.8, 16.7)	3.1 (0.6, 8.3)	3.2 (0.6, 8.4)	3.2 (0.6, 8.3)	6.0 (1.8, 13.1)	6.6 (2.0, 14.3)	7.4 (2.2, 15.6)
15	9.9 (4.3, 18.6)	10.1 (4.4, 18.8)	10.4 (4.5, 19.3)	3.7 (0.7, 9.9)	3.8 (0.7, 9.8)	3.8 (0.7, 10.1)	7.4 (2.3, 15.9)	8.3 (2.5, 17.0)	9.3 (2.8, 18.6)
20	11.5 (5.0, 21.4)	11.8 (5.2, 21.8)	12.1 (5.3, 22.3)	4.4 (0.8, 11.8)	4.5 (0.8, 11.8)	4.5 (0.8, 11.8)	9.2 (2.9, 19.5)	10.2 (3.2, 20.8)	11.3 (3.6, 22.5)

© 2024 Lo AC et al. *JAMA Network Open.*

eTable 12. Thirty-Year Cumulative Incidence and 95% CIs (%) of Any Cardiac Disease, Coronary Artery Disease, and Heart Failure for Average 15-Year-Old Hispanic Female With Dexrazoxane According to Cumulative Anthracycline Dose and Mean Cardiac Radiotherapy Dose

15 year old Hispanic female	Cumulative Anthracycline Dose (mg/m2)								
	Any Cardiac Disease			Coronary Artery Disease			Heart Failure		
Mean cardiac RT dose, Gy	200 mg/m2	250 mg/m2	300 mg/m2	200 mg/m2	250 mg/m2	300 mg/m2	200 mg/m2	250 mg/m2	300 mg/m2
0	4.9 (2.2, 9.3)	5.0 (2.2, 9.5)	5.2 (2.3, 9.8)	1.8 (0.4, 4.6)	1.9 (0.4, 4.6)	1.9 (0.4, 4.6)	2.8 (0.8, 6.1)	3.1 (0.9, 6.7)	3.5 (1.0, 7.4)
5	5.7 (2.5, 10.8)	5.9 (2.6, 11.1)	6.1 (2.7, 11.2)	2.2 (0.4, 5.4)	2.3 (0.5, 5.4)	2.3 (0.5, 5.4)	3.5 (1.0, 7.6)	3.9 (1.2, 8.3)	4.3 (1.3, 9.1)
10	6.7 (3.0, 12.5)	6.9 (3.1, 12.8)	7.1 (3.2, 13.1)	2.7 (0.5, 6.4)	2.7 (0.5, 6.4)	2.7 (0.5, 6.4)	4.4 (1.4, 9.6)	4.8 (1.5, 10.4)	5.4 (1.7, 11.1)
15	7.8 (3.6, 14.6)	8.1 (3.7, 15.0)	8.3 (3.8, 15.3)	3.2 (0.6, 7.6)	3.2 (0.7, 7.6)	3.2 (0.7, 7.6)	5.5 (1.8, 11.7)	6.0 (2.1, 12.5)	6.7 (2.3, 13.6)
20	9.2 (4.2, 17.1)	9.5 (4.4, 17.4)	9.8 (4.5, 17.7)	3.8 (0.8, 9.2)	3.8 (0.8, 9.3)	3.9 (0.8, 9.3)	6.8 (2.3, 14.3)	7.5 (2.6, 15.4)	8.3 (2.9, 16.6)

© 2024 Lo AC et al. *JAMA Network Open.*

eTable 13. Thirty-Year Cumulative Incidence and 95% CIs (%) of Any Cardiac Disease, Coronary Artery Disease, and Heart Failure for Average 15-Year-Old White Male With Dexrazoxane According to Cumulative Anthracycline Dose and Mean Cardiac Radiotherapy Dose

15 year old white male	Cumulative Anthracycline Dose (mg/m2)								
	Any Cardiac Disease			Coronary Artery Disease			Heart Failure		
Mean cardiac RT dose, Gy	200 mg/m2	250 mg/m2	300 mg/m2	200 mg/m2	250 mg/m2	300 mg/m2	200 mg/m2	250 mg/m2	300 mg/m2
0	5.4 (2.4, 9.9)	5.5 (2.5, 10.0)	5.7 (2.6, 10.1)	3.3 (0.7, 8.0)	3.4 (0.7, 8.1)	3.4 (0.7, 8.1)	1.4 (0.4, 3.3)	1.5 (0.5, 3.5)	1.7 (0.5, 3.9)
5	6.3 (2.9, 11.5)	6.5 (3.0, 11.6)	6.7 (3.0, 11.9)	4.0 (0.8, 9.4)	4.0 (0.8, 9.5)	4.1 (0.9, 9.4)	1.7 (0.5, 4.0)	1.9 (0.6, 4.4)	2.2 (0.7, 4.9)
10	7.4 (3.4, 13.3)	7.6 (3.4, 13.6)	7.8 (3.5, 13.9)	4.8 (1.0, 11.1)	4.8 (1.0, 11.0)	4.8 (1.0, 11.1)	2.2 (0.7, 4.9)	2.4 (0.8, 5.3)	2.7 (0.8, 5.8)
15	8.7 (3.9, 15.4)	8.9 (4.0, 15.8)	9.2 (4.1, 16.1)	5.7 (1.2, 13.2)	5.7 (1.2, 13.1)	5.8 (1.3, 13.2)	2.7 (0.9, 5.9)	3.1 (1.0, 6.4)	3.4 (1.1, 7.1)
20	10.2 (4.7, 18.2)	10.4 (4.8, 18.4)	10.7 (4.9, 18.7)	6.8 (1.4, 15.6)	6.8 (1.4, 15.8)	6.8 (1.4, 16.0)	3.4 (1.1, 7.3)	3.8 (1.2, 8.0)	4.3 (1.4, 8.9)

© 2024 Lo AC et al. *JAMA Network Open.*

eTable 14. Thirty-Year Cumulative Incidence and 95% CIs (%) of Any Cardiac Disease, Coronary Artery Disease, and Heart Failure for Average 15-Year-Old Black Male With Dexrazoxane According to Cumulative Anthracycline Dose and Mean Cardiac Radiotherapy Dose

15 year old black male	Cumulative Anthracycline Dose (mg/m2)								
	Any Cardiac Disease			Coronary Artery Disease			Heart Failure		
Mean cardiac RT dose, Gy	200 mg/m2	250 mg/m2	300 mg/m2	200 mg/m2	250 mg/m2	300 mg/m2	200 mg/m2	250 mg/m2	300 mg/m2
0	6.4 (2.7, 12.4)	6.6 (2.8, 12.7)	6.8 (2.9, 12.9)	3.7 (0.7, 9.5)	3.7 (0.7, 9.7)	3.8 (0.7, 9.7)	1.9 (0.5, 4.8)	2.1 (0.6, 5.3)	2.4 (0.6, 5.9)
5	7.5 (3.3, 14.5)	7.7 (3.4, 14.7)	7.9 (3.4, 15.1)	4.4 (0.8, 11.4)	4.4 (0.8, 11.6)	4.5 (0.9, 11.6)	2.4 (0.7, 5.9)	2.6 (0.7, 6.4)	3.0 (0.8, 7.1)
10	8.7 (3.8, 16.8)	9.0 (3.9, 17.2)	9.2 (4.0, 17.4)	5.3 (0.9, 13.5)	5.3 (1.0, 13.5)	5.4 (1.0, 13.7)	3.0 (0.9, 7.2)	3.3 (1.0, 7.8)	3.7 (1.1, 8.7)
15	10.2 (4.4, 19.3)	10.5 (4.6, 19.5)	10.8 (4.8, 20.0)	6.3 (1.1, 16.0)	6.4 (1.2, 16.1)	6.4 (1.2, 16.0)	3.7 (1.1, 8.8)	4.1 (1.2, 9.5)	4.6 (1.4, 10.4)
20	12.0 (5.2, 22.1)	12.2 (5.4, 22.4)	12.6 (5.5, 22.9)	7.5 (1.3, 18.8)	7.5 (1.3, 18.9)	7.6 (1.3, 18.8)	4.7 (1.5, 11.0)	5.2 (1.7, 11.7)	5.8 (1.9, 13.0)

© 2024 Lo AC et al. *JAMA Network Open.*

eTable 15. Thirty-Year Cumulative Incidence and 95% CIs (%) of Any Cardiac Disease, Coronary Artery Disease, and Heart Failure for Average 15-Year-Old Hispanic Male With Dexrazoxane According to Cumulative Anthracycline Dose and Mean Cardiac Radiotherapy Dose

15 year old Hispanic male	Cumulative Anthracycline Dose (mg/m2)								
	Any Cardiac Disease			Coronary Artery Disease			Heart Failure		
Mean cardiac RT dose, Gy	200 mg/m2	250 mg/m2	300 mg/m2	200 mg/m2	250 mg/m2	300 mg/m2	200 mg/m2	250 mg/m2	300 mg/m2
0	5.0 (2.3, 9.7)	5.2 (2.4, 9.9)	5.4 (2.5, 10.1)	3.2 (0.7, 7.7)	3.3 (0.7, 7.7)	3.3 (0.7, 7.9)	1.4 (0.4, 3.3)	1.5 (0.4, 3.6)	1.7 (0.5, 4.0)
5	5.9 (2.8, 11.2)	6.1 (2.9, 11.3)	6.3 (3.0, 11.7)	3.9 (0.8, 9.2)	3.9 (0.9, 9.2)	4.0 (0.9, 9.2)	1.7 (0.5, 4.1)	1.9 (0.6, 4.4)	2.2 (0.6, 4.9)
10	7.0 (3.3, 13.0)	7.2 (3.4, 13.3)	7.5 (3.5, 13.6)	4.6 (1.0, 10.9)	4.7 (1.0, 10.9)	4.7 (1.0, 11.0)	2.2 (0.7, 4.9)	2.4 (0.7, 5.4)	2.7 (0.8, 6.0)
15	8.2 (3.9, 15.1)	8.5 (4.0, 15.5)	8.7 (4.1, 15.9)	5.6 (1.2, 13.2)	5.6 (1.2, 13.1)	5.6 (1.2, 13.0)	2.7 (0.8, 6.1)	3.0 (0.9, 6.7)	3.4 (1.0, 7.4)
20	9.7 (4.5, 17.8)	9.9 (4.7, 18.3)	10.2 (4.9, 18.7)	6.5 (1.4, 15.3)	6.6 (1.4, 15.4)	6.7 (1.5, 15.7)	3.4 (1.1, 7.7)	3.8 (1.2, 8.4)	4.2 (1.3, 9.2)

© 2024 Lo AC et al. *JAMA Network Open.*

eFigure 1. Cumulative Incidence of Any Cardiac Disease Based on Mean Heart Dose for Average 15-Year-Old Patient. A: Doxorubicin=200mg/m^2 with dexrazoxane; B: Doxorubicin=250mg/m^2 with dexrazoxane; C: Doxorubicin=300mg/m^2 with dexrazoxane.

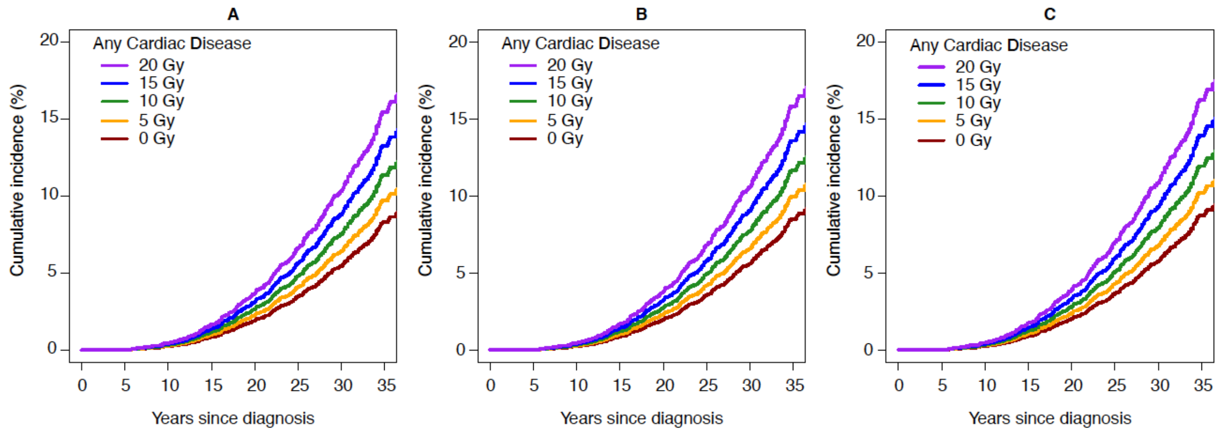

© 2024 Lo AC et al. *JAMA Network Open.*

eFigure 2. Thirty-Year Cumulative Incidence of Any Cardiac Disease in the Average 12-Year-Old Female on Each Trial. S2-A: AHOD0031 standard arm (85% mediastinal RT); S2-B: AHOD0031 experimental arm (40% mediastinal RT); S2-C: AHOD0831; S2-D: AHOD1331; S2-E: S1826.

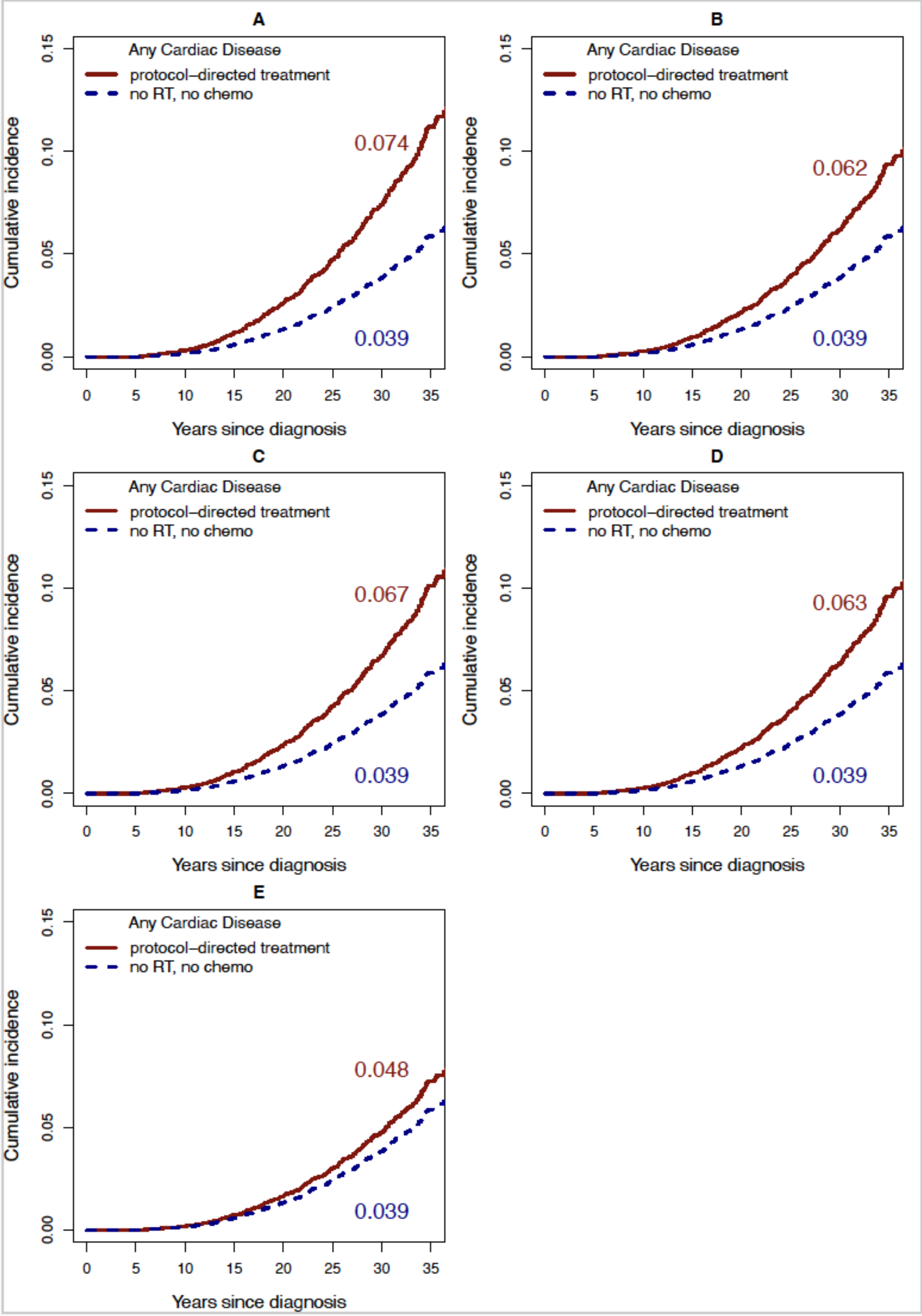

© 2024 Lo AC et al. *JAMA Network Open.*
