# Supplementary material for: Late Cardiac Toxic Effects Associated With Treatment Protocols for Hodgkin Lymphoma in Children
Source: JAMA Netw Open. 2024 Jan 19;7(1):e2351062. doi: 10.1001/jamanetworkopen.2023.51062 (PMC10799264; doi:10.1001/jamanetworkopen.2023.51062)
Supplement: Supplement 2. — Data Sharing Statement [file jamanetwopen-e2351062-s002.pdf]

Data Sharing Statement

Lo. Late Cardiac Toxic Effects Associated With Treatment Protocols for Hodgkin Lymphoma in Children. *JAMA Netw Open*. Published January 19, 2024.
doi:10.1001/jamanetworkopen.2023.51062

Data
Data available: No

Additional Information
Explanation for why data not available: We need to abide by the COG and CCSS data policies. The Children's Oncology Group data underlying this article can be requested at datarequest@childrensoncologygroup.org. The CCSS data underlying this article can be requested at https://ccss.stjude.org.
